# The Effects of Smoking on the Severity of Atopic Dermatitis in Saudi Arabia

**DOI:** 10.7759/cureus.50315

**Published:** 2023-12-11

**Authors:** Basma A Alturki, Rahaf Almutairi, Atheer G Al-mutairi, Danah Alrajhi, Faris H Binyousef, Fajer Alzamil

**Affiliations:** 1 Dermatology, College of Medicine, Imam Mohammad Ibn Saud Islamic University, Riyadh, SAU; 2 Dermatology, Unaizah College of Medicine and Medical Sciences, Qassim University, Unaizah, SAU; 3 Ophthalmology, King Khaled Eye Specialist Hospital, Riyadh, SAU

**Keywords:** skin, allergy, dermatitis, cigarettes, eczema

## Abstract

Background

Atopic dermatitis (AD) is a well-known inflammatory skin disease that is associated with a family history of other atopic diseases. Tobacco smoking has been found to affect AD as well as several other inflammatory skin diseases. In this study, we aimed to investigate this association and to elucidate the link between dose-dependent tobacco exposure and symptom severity.

Methods

This cross-sectional study was conducted on individuals from the general population of Riyadh, Saudi Arabia. Data were collected using an online questionnaire. All statistical analyses were performed using RStudio, version 1.1.363 (RStudio, PBC, Boston, Massachusetts, United States). Questions about the participants’ age, sex, and occupational status were included. The participants were asked to report their daily handwashing habits and history of atopic diseases. Data on the smoking duration, number of cigarettes smoked per day, and passive exposure were collected.

Results

A total of 510 participants (41.3 %) reported having AD. Smoking was significantly associated with an increased prevalence of AD. The odds of having AD were 1.78 and 2.27 times higher in occasional smokers (odds ratio (OR) = 1.78, p < 0.05) and daily smokers (OR = 2.27, p < 0.001) than in non-smokers. Neither smoking frequency (p = 0.19) nor duration (p = 0.73) was significantly associated with AD prevalence.

Conclusion

Smoking is significantly associated with an increased prevalence of AD. Adults should be discouraged from smoking in order to prevent adult-onset AD. The level of nicotine exposure should be measured objectively in future studies.

## Introduction

Atopic dermatitis (AD) is a well-known inflammatory skin disease that is associated with a family history of other atopic diseases. AD commonly occurs in early childhood, but symptoms often begin in adulthood, resulting in many detrimental consequences, such as work loss [1]. The prevalence of AD in Saudi Arabia has been reported to be 35.9% [2]. Over the past five years, there has been a sudden increase in the number of AD cases in many countries. This number continues to grow [3], reflecting the importance of identifying the risk factors that can lead to AD. One of these risk factors is gene mutations, such as the filaggrin gene mutation [4-6]. Another risk factor is infectious agents, such as *Staphylococcus aureus* [7,8]. Finally, AD is also associated with exogenous and lifestyle factors, such as wet work, contact with irritant material, and cigarette smoking. All the aforementioned factors have been reported to play a role in AD pathogenesis [8-12].

AD, as well as several other inflammatory skin diseases, such as systemic lupus erythematosus, can be affected by tobacco smoking [13], and the association between the two has been proven by several studies [14-16]. Smoking can not only affect the inflammatory process through cytokine activity and oxidative stress but can also mediate active hand dermatitis and hinder the normal healing process [17,18]. Many studies have reported different AD-related results between active and passive smoking. In the case of active smoking and adult-onset AD, the results are diverse [19]. However, in the case of passive smoking, which is exposure to environmental tobacco smoke during early childhood, it was noted that children were more likely to develop AD later in life [20,21]. With this in mind, the present study aimed to investigate such associations as well as to elucidate the link between dose-dependent tobacco exposure and the severity of AD symptoms. 

## Materials and methods

Study design

The present cross-sectional study was conducted over a period of four months in Riyadh, Saudi Arabia. This study aimed to investigate the association between AD and smoking habits. Data were collected using an online questionnaire. The study was approved by the Institutional Review Board Committee of Imam Mohammed Ibn Saud Islamic University, Riyadh, Saudi Arabia (approval number: HAPO-01-R-001). Consent was obtained from all the participants, and all personal information was kept confidential.

The questionnaire was divided into three sections. The first section collected the respondents' demographic information (age, sex, occupational status, comorbidities, and family history). The second section collected information on smoking habits (tobacco products used, frequency, and duration of smoking). The participants were asked if they were previously diagnosed with AD. The last section collected information regarding AD and its severity, which was assessed using the Patient-Oriented Eczema Measure (POEM) questionnaire. The POEM questionnaire includes seven Likert-scale items. Each item is scored on a scale from 0 (none of the days) to 4 (daily). For each respondent, the total score is calculated by summing the scores of the seven items; the maximum possible score is 28. The following cutoff points were used to grade the severity of AD: 0 to 2, clear or almost clear; 3 to 7, mild AD; 8 to 16, moderate AD; 17 to 24, severe AD; and 25 to 28, very severe AD. The questionnaire was validated through a pilot study that included around 15 participants, which was conducted to identify any question- and language-related issues and the scope for modifying or improving the content.

Statistical analysis

Statistical analyses were performed using RStudio, version 1.1.363 (RStudio, PBC, Boston, Massachusetts, United States). Counts and percentages were used to summarize the distribution of categorical variables. The mean ± standard deviation was used to summarize the distribution of continuous variables. The chi-square test was used to assess the associations between categorical variables. Spearman's correlation coefficient was used to assess the association between continuous or ordinal variables. The Mann-Whitney and Kruskal-Wallis tests were used to compare POEM scores between the two groups. Cronbach's alpha was used to assess the reliability of the POEM items, with a lower acceptable bound of 0.7. Binary logistic regression was used to assess the association between smoking and the incidence of AD after adjusting for sex, age, marital status, exercise frequency, family history of allergic diseases, and allergic comorbidities (food allergy, allergic rhinitis, and asthma). A p-value lower than 0.05 was considered statistically significant for all analyses.

## Results

The study questionnaire was completed by 1235 respondents, of which 690 (55.9%) were male and 545 (44.1%) were female. Respondents aged 18-35 years accounted for the majority of the study sample (n=869, 70.4%), followed by those aged 36-55 years (n=271, 22.9%). More than half of the study participants were non-smokers (n=710, 57.5%), while 345 (27.9%) of them smoked daily. Only 74 (5.99%) and 106 (8.58%) participants were ex-smokers and occasional smokers, respectively. Regarding marital status, 832 (67.4%) respondents were single, and 380 (30.8%) were married. The demographic characteristics of the participants are presented in Table 1.

**Table 1:** Demographics of study participants

	N (%)
Sex:	
Female	545 (44.1%)
Male	690 (55.9%)
Age:	
< 18 years	57 (4.62%)
18–35 years	869 (70.4%)
36–55 years	271 (22.9%)
> 55 years	38 (3.08%)
Smoker:	
No	710 (57.5%)
Ex-smoker	74 (5.99%)
Sometimes	106 (8.58%)
Daily	345 (27.9%)
Marital status:	
Single	832 (67.4%)
Divorced	16 (1.30%)
Married	380 (30.8%)
Widowed	7 (0.57%)
Employment:	
Farmer	2 (0.16%)
Health care provider	63 (5.10%)
Labour job	10 (0.81%)
Office job	259 (21.0%)
Other	144 (11.7%)
Student	601 (48.7%)
Unemployed	156 (12.6%)
Exercise days/week:	
Never	656 (53.1%)
1–2 day	215 (17.4%)
3–5 days	286 (23.2%)
Daily	78 (6.32%)
Daily frequency of handwashing with soap and water:	
< 10 times	837 (67.8%)
10–20 times	324 (26.2%)
> 20 times	74 (5.99%)
Time taken to wash hands with soap and water:	
< 10 s	480 (38.9%)
10–20 s	537 (43.5%)
20–40 s	175 (14.2%)
> 40 s	43 (3.48%)
Daily smoking frequency:	
< 10 cigarettes	143 (31.7%)
11–20 cigarettes	235 (52.1%)
21–40 cigarettes	64 (14.2%)
> 40 cigarettes	9 (2.0%)
Smoking duration:	
< 10 years	231 (51.2%)
10–20 years	107 (23.7%)
> 20 years	113 (25.1%)
Smoked at least 100 cigarettes in a lifetime:	
No	93 (20.6%)
Yes	358 (79.4%)

Half of the respondents were students (n=601, 48.7%), and a quarter had office jobs (n=259, 21%). Unemployed respondents represented 12.6% (n=156) of the study sample. Half of the respondents did not exercise, while the remaining 17.4% (n=215), 23.2% (n=286), and 6.32% (n=78) exercised one to two times a week, three to five times a week, and daily, respectively. A quarter of the respondents washed their hands 10-20 times/day. Hand washing time varied between respondents, with 38.9% (n=480) and 43.5% (n=537) of the respondents reporting washing their hands for < 10 seconds and 10-20 seconds, respectively. Only 14.2% (n=175) and 3.48% (n=43) reported washing their hands for 20-40 seconds and > 40 seconds, respectively. Half of the respondents smoked 10-20 cigarettes daily, and 31.7% (n=143) smoked < 10 cigarettes. Regarding the smoking duration reported by current smokers (smoked sometimes or daily), half reported that they had smoked for < 10 years and a quarter reported that they had smoked for 10-20 years. More than three-quarters of the respondents (n=358, 79.4%) had smoked at least 100 cigarettes in their lifetime.

**Figure 1:**
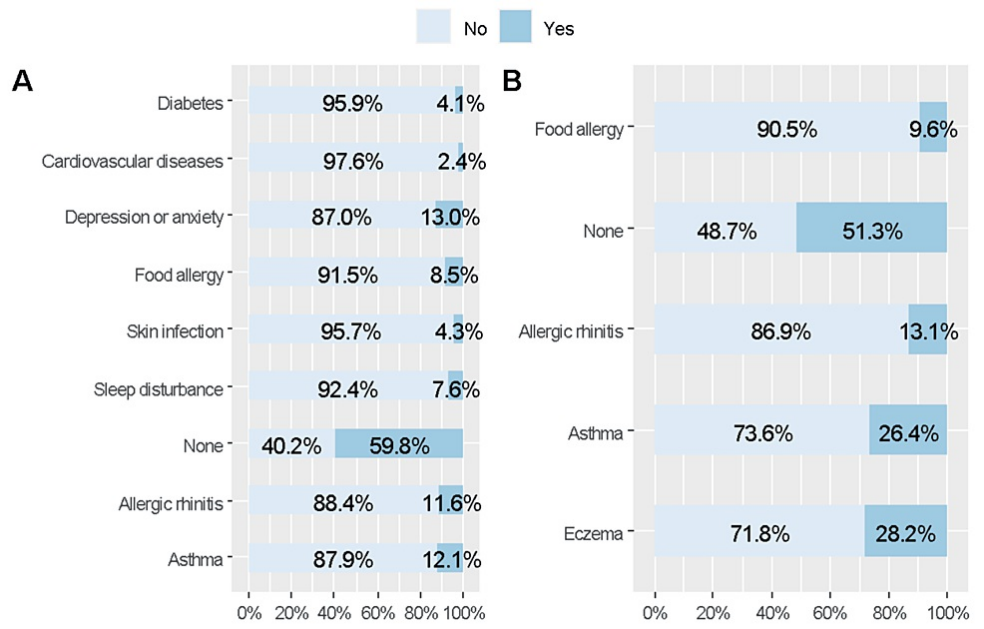
Comorbidities and family history of allergic conditions in the included respondents (A) Personal comorbidities. (B) Family history

More than half of the respondents did not have comorbidities (59.8%) or did not report any family history of allergic conditions (51.3%). The most commonly reported comorbidities were asthma (12.1%) and allergic rhinitis (11.6%). A quarter of the respondents had a family history of either AD (28.2%) or asthma (26.4%), while 13.1% reported a family history of allergic rhinitis (Figure 1).

Slightly less than half of the respondents (n=510, 41.3 %) reported being diagnosed with AD. Of the participants diagnosed with AD, 47.6% (n=243) were smokers. Of these, 11.5% (n=28) reported that AD worsened when smoking, 39.5% (n=96) were unsure, and 49.0% (n=119) did not think so. The average POEM score was 9.51 ± 7.46. According to the POEM scores, one-third of the respondents had moderate AD (n=183, 35.9%), 23.9% (n=122) had mild AD, and 21.8% (n=111) had clear AD. The remaining 14.5% (n=74) and 3.92% (n=20) of patients had severe and very severe AD, respectively (Table 2).

**Table 2:** Prevalence of atopic dermatitis and its severity POEM, Patient-Oriented Eczema Measure; AD, Atopic Dermatitis

	N (%)
Been diagnosed with AD:	
No	725 (58.7%)
Yes	510 (41.3%)
Smoked before:	
No	267 (52.4%)
Yes	243 (47.6%)
AD gets worse when smoking:	
Maybe	96 (39.5%)
No	119 (49.0%)
Yes	28 (11.5%)
POEM classification:	
Clear	111 (21.8%)
Mild	122 (23.9%)
Moderate	183 (35.9%)
Severe	74 (14.5%)
Very severe	20 (3.92%)
Average POEM score	9.51 ± 7.46

**Figure 2:**
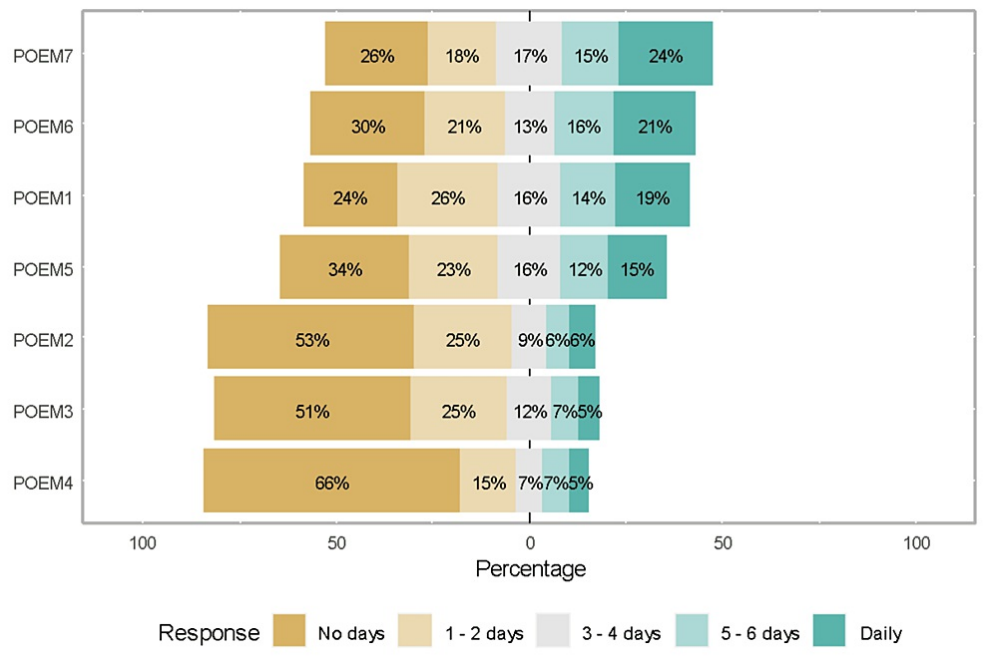
Responses to POEM questionnaire items POEM, Patient-Oriented Eczema Measure

Figure 2 shows the responses to individual POEM questionnaire items. Skin flaking and dryness were the most troublesome items (items 6 and 7), whereas weeping (item 4) and bleeding (item 3) were the least reported. The other items were itching (item 1), sleep disturbance (item 2), and skin cracking (item 5).

Table 3 elucidates the association between smoking and AD; the results showed that smoking was significantly associated with the prevalence of AD, with only 37.6% (267) of non-smokers reporting AD compared with 47% (n=162) of daily smokers (p < 0.05). The prevalence of AD among ex-smokers and occasional smokers was similar. Neither smoking frequency (p = 0.19) nor duration (p = 0.73) was significantly associated with AD prevalence. The daily frequency of handwashing was significantly associated with the prevalence of AD (p = 0.002); there was an increasing trend in the prevalence of AD with an increase in the daily frequency of handwashing. The time taken to wash the hands with soap and water was not significantly associated with the prevalence of AD (p = 0.649).

**Table 3:** Association between smoking and atopic dermatitis The data is represented as N (%)

	No, N (%)	Yes, N (%)	Overall p-value
Smoking status:			0.023
No	443 (62.4%)	267 (37.6%)	
Ex-smoker	41 (55.4%)	33 (44.6%)	
Sometimes	58 (54.7%)	48 (45.3%)	
Daily	183 (53.0%)	162 (47.0%)	
Daily smoking frequency:			0.190
< 10 cigarettes	73 (51.0%)	70 (49.0%)	
11–20 Cigarettes	133 (56.6%)	102 (43.4%)	
21–40 cigarettes	33 (51.6%)	31 (48.4%)	
> 40 cigarettes	2 (22.2%)	7 (77.8%)	
Smoking duration:			0.730
< 10 years	124 (53.7%)	107 (46.3%)	
10–20 years	54 (50.5%)	53 (49.5%)	
> 20 years	63 (55.8%)	50 (44.2%)	
Daily frequency of handwashing with soap and water:			0.002
< 10 times	515 (61.5%)	322 (38.5%)	
10–20 times	179 (55.2%)	145 (44.8%)	
> 20 times	31 (41.9%)	43 (58.1%)	
Time taken to wash hands with soap and water:			0.649
< 10 s	278 (57.9%)	202 (42.1%)	
10–20 s	311 (57.9%)	226 (42.1%)	
20–40 s	108 (61.7%)	67 (38.3%)	
> 40 s	28 (65.1%)	15 (34.9%)	

The results showed that longer smoking duration was associated with a lower POEM score (r = -0.136, p < 0.005) and lower POEM class (r = -0.145, p < 0.05). None of the remaining variables were significantly associated with the severity of AD (Table 4). 

**Table 4:** Association between atopic dermatitis severity and smoking POEM, Patient-Oriented Eczema Measure; AD, Atopic Dermatitis The data is represented as p-value ^*^p < 0.05

	Smoker	Smoking duration	Smoking frequency	Hand washing frequency	Hand washing duration
POEM	-0.061	-0.136^*^	0.042	0.111	-0.043
POEM class	-0.059	-0.145^*^	0.029	0.095	-0.071

Table 5 presents the results of the binary logistic regression analysis that was used to assess whether smoking was significantly associated with the incidence of AD after adjustments for sociodemographic characteristics. The results showed that the association between smoking and AD remained significant after adjustments for age, sex, and marital status. The odds of having AD were 2.09 times higher in ex-smokers than in non-smokers (odds ratio (OR) = 2.09, p < 0.05). The odds of having AD were 1.78 and 2.27 times higher in occasional smokers (OR = 1.78, p < 0.05) and daily smokers (OR = 2.27, p < 0.001) than in non-smokers. A family history of allergic conditions was associated with higher odds of developing AD (OR = 3.3, p < 0.001). The odds of developing AD were 1.61 times and 3.4 times higher in respondents who suffered from allergic rhinitis (OR = 1.61, p < 0.05) and food allergy (OR = 3.4, p < 0.001) than in respondents who did not. The odds of developing AD were lower in males than in females (OR = 0.56, p < 0.001). None of the remaining factors were significantly associated with the incidence of AD.

**Table 5:** Binary logistic regression analysis for factors associated with atopic dermatitis

Predictors	Odds ratios	Confidence interval	p-value
(Intercept)	1.17	0.61–2.24	0.633
Sex: Male vs. Female	0.56	0.40–0.77	< 0.001
Age: < 18 years	Ref		
Age: 18–35 years	0.96	0.52–1.81	0.906
Age: 36–55 years	1.20	0.57–2.56	0.624
Age: > 55 years	0.56	0.20–1.53	0.260
Smoker: Non-smoker	Ref		
Smoker: Ex-smoker	2.09	1.19–3.67	0.010
Smoker: Sometimes	1.78	1.11–2.84	0.016
Smoker: Daily	2.27	1.59–3.25	< 0.001
Marital status: Single	Ref		
Marital status: Divorced	0.59	0.17–1.79	0.369
Marital status: Married	1.24	0.83–1.83	0.288
Marital status: Widowed	0.44	0.06–2.34	0.359
Exercise	0.94	0.83–1.07	0.330
Family history: Yes vs. No	3.3	2.56–4.35	< 0.001
Comorbidities			
Asthma: Yes vs. No	0.82	0.55–1.21	0.313
Allergic rhinitis: Yes vs. No	1.61	1.08–2.41	0.019
Food allergy: Yes vs. No	3.40	2.15–5.50	< 0.001

## Discussion

In concordance with previous studies, this study revealed a remarkable association between smoking and AD [17-27]. In previous studies, this relationship appeared to be significant whether it was subjectively reported or objectively measured [27]. In our study, the prevalence of AD among 1235 respondents from the general population was 41.3% (n=510), of whom 47% (n=162) reported smoking daily. A significant association between smoking and AD prevalence was also detected among ex-smokers and occasional smokers. Similarly, a meta-analysis of observational studies revealed a high prevalence of AD and smoking in both adults and children [28]. Furthermore, a systematic review was performed on 20 studies found in the PubMed database, which assessed the relationship between tobacco smoking and AD [29]. Among these studies, eight included population-based surveys and scientific investigations (seven were cross-sectional and one was a cohort study), and four found a positive correlation between smoking and AD. However, no association was found in the remaining four studies. On the other hand, a study published in the British Journal of Dermatology included corresponding controls and three occupational cohorts and concluded that a smaller number of AD cases was reported among smokers, while a larger number of cases was reported among non-smokers (437 and 1294 participants, respectively out of 13 452) [30]. 

In the present study, higher consumption of tobacco did not affect the prevalence of AD, as neither smoking frequency (p = 0.19) nor duration (p = 0.73) was significantly associated with the prevalence of AD. By contrast, an American study, which included a sample of 25,428 individuals, investigated the dose-dependent relationship between AD and nicotine exposure and reported higher odds of having active AD and, consequently, significantly increased AD prevalence among individuals consuming larger amounts of tobacco [17].

The severity of AD was measured by integrating the POEM score into the questionnaire in order to perform an accurate assessment and to avoid subjective bias; the average score obtained was 9.51 ± 7.4. Additionally, the questionnaire categorized the participants, who reported having AD, into five subgroups: very severe, severe, moderate, mild, and clear AD groups; this revealed that the moderate AD group accounted for one-third of the study population. Besides, the correlation between smoking duration and frequency and AD severity was assessed using the Spearman method with listwise deletion, which showed that a lower POEM score was associated with longer smoking duration (r = -0.136, p < 0.005) and lower POEM class (r = -0.145, p < 0.05). However, a Danish study analyzed data from a private dermatology practice in order to assess the severity of AD in 522 consecutive patients; the results revealed no significant correlation in 224 current smoking patients based on a severity scoring system with scores ranging from 0 to 3 [27]. This earlier study included multiple parameters, such as erythema, vesicles, scaling, pruritus, area, and fissures.

To the best of our knowledge, most of the studies that evaluated the association between tobacco smoking and hand AD were conducted in Europe and the United States. This was the first primary study on this topic conducted in Saudi Arabia. The strength of this study was that a validated method, involving the POEM questionnaire, was used to estimate AD severity based on self-reported surveys. However, this study had a few limitations. First, the data were collected using an online questionnaire. Second, the study sample was from the general population and was not focused on patients with AD, which can narrow the scope of our findings. Third, a self-reported survey is not sufficient for assessing the severity of AD and the effects of nicotine exposure; this can be addressed by including more reliable measured variables for evaluating the relationship between the serum nicotine concentration and the severity of AD symptoms. 

## Conclusions

The results of the present study reveal that tobacco smoking is significantly associated with the presence of AD; however, this association was not detected in the case of smoking frequency or duration. It is important to consider smoking cessation for the prevention and management of AD, as well as while designing multiple educational programs and community campaigns regarding the various factors that affect AD. Future studies with objective assessments of tobacco exposure and AD are needed to address this dose-dependent association further.
